# Anaesthesia as an influence in tumour progression

**DOI:** 10.1007/s00423-021-02078-z

**Published:** 2021-02-01

**Authors:** Jadie Plücker, Naita M. Wirsik, Alina S. Ritter, Thomas Schmidt, Markus A. Weigand

**Affiliations:** 1 Department of Anaesthesiology, University of Heidelberg, Heidelberg, Germany; 2 Department of General, Visceral and Transplantation Surgery, University of Heidelberg, Heidelberg, Germany

**Keywords:** General anaesthesia, Cancer recurrence, Metastasis, Narcotics, Opioids, Regional anaesthesia

## Abstract

**Purpose:**

Tumour growth and the formation of metastases are essential elements in the progression of cancer. The centre of treatment is the surgical resection of primary solid tumours. But even if the tumour can be removed without microscopic residual cells, local recurrences and distant metastases occur and determine the patient’s fate. During the operation, tumour cells are shed from the primary tumour and released into the circulation. These circulating tumour cells might play an important role in the formation of new tumour sites. Therefore, a functional innate and adaptive immune system is essential, especially in this perioperative period. Anaesthesia influences consciousness and pain perception and interacts directly with the immune system and tumour cells.

**Methods:**

Review of the current literature concerning intra- and postoperative anaesthetic decisions and tumour progression.

**Results:**

There are beneficial aspects for patient survival associated with total intravenous anaesthesia, the use of regional anaesthetics and the avoidance of allogeneic red blood cell transfusions. Alternatives such as irradiated intraoperative blood salvage and preoperative iron supplementation may be advantageous in cases where transfusions are limited or not wanted. The immunosuppressive properties of opioids are theoretical, but strong evidence to avoid them does not exist. The application of nonsteroidal anti-inflammatory drugs and postoperative nausea and vomiting prophylaxis do not impair the patient’s survival and may even have a positive effect on tumour regression.

**Conclusion:**

Anaesthesia does play an important part in the perioperative period in order to improve the cancer-related outcome. Further research is necessary to make more concrete recommendations.

## Anaesthesia as a part of perioperative medicine

The focus of modern medicine is to see the whole, individual patient. It is an interdisciplinary discussion, not only about the disease, but also about the health, physical abilities und social integrity of patients altogether. The numerous disciplines of medicine and the areas of current research interact on a daily basis to gain new insights for curative treatments, recovery plans, prevention and palliative care. The elements of anaesthesia, including narcosis, analgesia and generally perioperative care, are necessary to make many of these aims possible.

After centuries of developments and improvements in medical procedures, hygiene, infrastructure and technologies, cancer is now one of the biggest health challenges. For almost any type of solid tumour, the only curable treatment option is surgery. But chemotherapy, radiation, immunotherapy and other new strategies help to improve survival rates, recovery outcomes and quality of life. The operation of a tumour is probably the most critical part of this equation. The role of modern anaesthesia is not only to make these surgeries possible for almost everyone of all ages and general health conditions but also to influence the course of cancer outcome itself. Current research highlights the effects of anaesthesia on tumour cells, their microenvironment and the immune system. Therefore, a general influence of the anaesthetic technique, including the choice of narcotics, pain management, patient blood management, oxygen saturation and postoperative nausea and vomiting (PONV) prophylaxis, may be important to understand and probably alter the interaction between the tumour cells and the patient’s organism, thereby improving the outcome of treatment.

## Choice of narcotics

In the past decades, scientists searched for new narcotics with increased potential and reduced side effects. Today, there are numerous substances to induce and maintain narcosis. The two most commonly used drugs are propofol and sevoflurane. Propofol is used for intravenous induction and maintenance of short procedures or to prevent severe cases of nausea and vomiting (total intravenous anaesthesia [TIVA]). Sevoflurane as an inhalational agent is a potent and cheap way to maintain narcosis, with additional benefits for patients with cardiovascular health issues or for children as an inhalational induction. If the procedure or the condition of the patient does not dictate the choice of narcotics, the anaesthesiologist decides based on his preferences. Today, the decision for either a total intravenous or a balanced anaesthesia in cancer surgery is in question in terms of the possible impact on outcome.

**Fig. 1:**
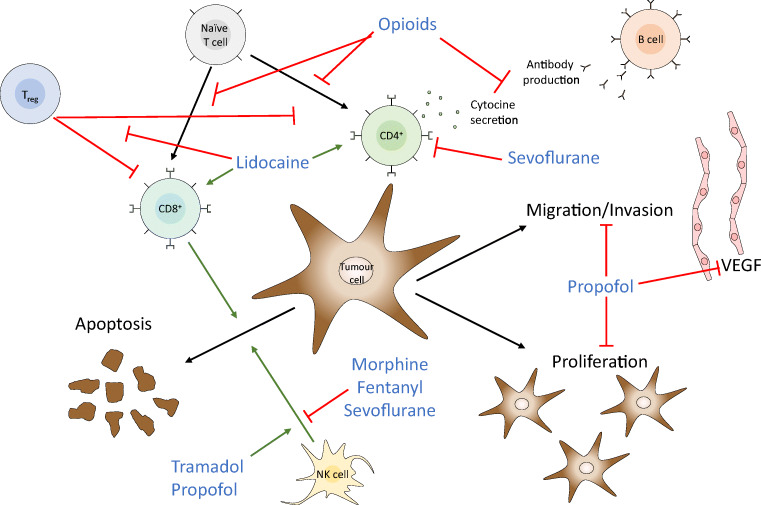
Exemplary illustration of the interaction of anaesthetic agents with the tumour environment and the immunological response. Graphics program: MS PowerPoint

In vitro experiments with different types of cancer cells can detect direct and indirect antitumour effects of propofol. Some of the direct effects include inhibition of proliferation, migration and invasion and induction of apoptosis based on micro-RNA alterations (e.g. induction of miR-125a-5p, miRNA-133a, etc.) and an influence on signalling pathways such as the inhibition of mitogen-activated protein kinase (MAPK), nuclear factor ‘kappa-light-chain-enhancer’ of activated B cells (NF-κB) and hypoxia-inducible factor-1 alpha (HIF-1α) [1–3]. Contrarily, propofol was also found to activate nuclear factor erythroid 2-related factor 2 (Nrf2) in gallbladder cancer, which leads to an inhibition of apoptosis [1]. Indirectly, propofol interferes with tumour progress due to increasing chemosensitivity and maintaining immunological function. Increased chemosensitivity was found for trastuzumab in breast cancer [4], paclitaxel and cisplatin in ovarian cancer [5, 6] and gemcitabine in pancreatic cancer [7]. Propofol preserves the immunological function compared to sevoflurane, which suppressed functional T1-lymphocytes in human colorectal and cervical cancer [8, 9] (Fig. 1).

These findings could be supported by in vivo data showing, for example, a reduced rate of lung metastasis of breast cancer cells after a single treatment with propofol in mice [10]. However, another in vivo study showed a depletion of tumour-associated macrophages from the tumour microenvironment and an upregulation of the immune checkpoint programmed death ligand-1 (PD-L1) by sevoflurane in melanomas, indicating a possible positive effect of sevoflurane in combination with the checkpoint inhibitor programmed death-1(PD-1) [11].

Clinical data are required to prove that these hypotheses are valid in humans. There are many retrospective analyses that indicate a beneficial effect of propofol versus inhalational agents. A meta-analysis by Jin et al. in 2019 summarized twelve studies with a pooled hazard ratio for all-cause mortality of 0.73 [95% CI 0.60, 0.89] for TIVA. But divided in subgroups of cancer types, only a statistical analysis of breast and colorectal cancer could be conducted, showing a positive trend for TIVA in colorectal but not in breast cancer. The limitations of this data are numerous: for instance, a retrospective study design, lack of statistical power and no clear control of confounding factors [12]. However, a large cohort study (166 966 inhalational, 29 337 TIVA) in Japan found no difference in overall survival in terms of any digestive cancer surgery, but a slight improvement in recurrence-free survival for TIVA when instrumental variable analyses were performed (HR 0.92, 95% CI 0.87, 0.98 *p* = 0.01) [13].

Prospective studies to compare the outcome of cancer surgery are rare. One of the few completed studies showed a significant reduction of vascular endothelial growth factor (VEGF) release in breast cancer surgery for TIVA with a positive trend, but no significant difference in short-term survival [14] (Fig. 1). Another study measured a preserved natural killer cell cytotoxicity also in breast cancer surgery, which supports the hypothesis of an immunoprotective effect of propofol [15]. A follow-up of survival data needs to be awaited. A third prospective study compared a TIVA in combination with a paravertebral nerve block with sevoflurane both in addition to remifentanil intraoperatively and a sufentanil pump postoperatively in radical lung resection for non-small cell lung cancer (NSCLC). Using a TIVA in combination with a paravertebral nerve block could reduce the release of VEGF and transformation growth factor-β (TGF-β) and improve the analgesic coverage for patients. Again, the follow-up regarding mortality rates is not yet ready [16]. A large (*n* = 2108) randomized controlled multicentre trial compared recurrence rates (7-year follow-up) after curative resection of breast cancer and found no difference between a paravertebral block in combination with propofol and general anaesthesia with sevoflurane and opioids [17] (Table 1).

**Table 1:** Prospective randomized controlled trials with survival/mortality as a primary or secondary endpoint

Name	Author	Year	Cancer type	Power	Result
Effects of propofol-/remifentanil-based total intravenous anaesthesia versus sevoflurane-based inhalational anaesthesia on the release of VEGF-C and TGF-β and prognosis after breast cancer surgery: a prospective, randomized and controlled study	Yan et al.	2018	Breast cancer	*n* = 80	- Significant reduction in VEGF-C in TIVA group (12 pg/ml) vs. (50 pg/ml) (*P* = 0.008) - No significant differences in TGF-β concentration - Two-year RFS rates were 78% and 95% in the SEV and TIVA groups (*P* = 0.221)
The effects of perioperative anaesthesia and analgesia on immune function in patients undergoing breast cancer resection: a prospective randomized study	Cho et al.	2017	Breast cancer	*n* = 48	- NKCC (%) increased in the propofol-ketorolac group (15.2 to 20.1, *P* = 0.048), whereas it decreased in the sevoflurane-fentanyl group (19.5 to 16.4, *P* = 0.032) - One patient in the sevo-fentanyl group had recurrence - No patient had metastasis in either group within 2 years after surgery
Recurrence of breast cancer after regional or general anaesthesia: a randomized controlled trial	Sessler et al.	2019	Breast cancer	*n* = 2108	- No reduction in recurrence of paravertebral block and propofol compared with sevoflurane and opioids (hazard ratio 0.97, 95% CI 0.74–1.28; *p* = 0.84)
Perioperative immunomodulatory therapy does not decrease postoperative recurrence rate of rectal cancer	Gan et al.	2015	Rectal cancer	*n* = 150	- Celecoxib, given 5 days pre- and post-surgery, reduces postoperative inflammation (CRP, IL6) - No effect on recurrence within 45 months (*p* = 0.549)
Impact of epidural analgesia on mortality and morbidity after surgery: systematic review and meta-analysis of randomized controlled trials	Pöpping et al.	2014	All surgical patients	*n* = 2201	- Risk of death was decreased with epidural analgesia (3.1% vs 4.9%; odds ratio, 0.60; 95% confidence interval, 0.39–0.93)
Effects of combined epidural and general anaesthesia on intraoperative hemodynamic responses, postoperative cellular immunity and prognosis in patients with gallbladder cancer: a randomized controlled trial	Zhu et al.	2017	Gallbladder cancer	*n* = 144	- Increased survival rates of CD^3+^, CD^4+^, and CD^4+^/CD^8+^ in combined epidural and general anaesthesia group in comparison to general anaesthesia alone - No difference in 1-year, 2-year and 3-year survival rates
Combined anaesthesia shows better curative effect and less perioperative neuroendocrine disorder than general anaesthesia in early-stage NSCLC patients	Pi et a.	2019	NSCLC	*n* = 149	- OS GA 52% vs. EGA 54.1% (*p* = 0.664) - DFS GA 20% vs. EGA 31% (*p* = 0.08) - Serum levels of IL-1, IL-8, hs-CRP and TNF-α were generally lower in the EGA group than in the GA group
Intraoperative autologous blood transfusion use during radical hysterectomy for cervical cancer: long-term follow-up of a prospective trial	Engle et al.	2012	Cervical cancer	*n* = 71	No difference in long-term survival (83% in both groups after 12 years)

*CI* confidence interval, *CRP* c-reactive protein, *DFS* disease-free survival, *EGA* combined epidural and general anaesthesia, *GA* general anaesthesia, *IL* interleukin, *NKCC* natural killer cell cytotoxicity, *NSCLC* non-small cell lung cancer, *OS* overall survival, *RFS* recurrence-free survival, *TGF-β* transformation growth factor β, *TIVA* total intravenous anaesthesia, *VEGF-C* vascular endothelium growth factor-C

The existing evidence is conflicting. On the one hand, experimental and many retrospective studies favour a propofol-based total intravenous anaesthesia to improve the outcome of surgery on certain cancer types such as colorectal cancer. On the other hand, for cancer types such as breast cancer, the choice of narcotics seems irrelevant. On this basis, no concrete recommendations can be made and prospective studies with a sufficient number of cases and long-term follow-up for other cancer types must be conducted.

## Pain management

Analgesia is an important factor in maintaining narcosis during a surgical procedure. But in cancer patients, pain is not only caused by wound healing but derives from the cancer itself. Therefore, pain treatment is also an issue before and after the surgery. Pain not only affects the physical and mental state of the patient but also alters the inflammatory and immunological responses of the body. According to the World Health Organization (WHO) cancer pain ladder, cancer pain treatment begins with nonopioids, followed by mild and severe opioids until the patient is pain free. There are numerous drugs within these categories with different potential to alleviate pain but also different effects towards tumour progression. For the perioperative period, modern anaesthesia also provides regional anaesthetic techniques such as an epidural catheter or nerve blocks with the potential not only to eliminate pain but also to improve cancer outcome.

### NSAIDs

Although nonopioids, such as nonsteroidal anti-inflammatory drugs (NSAIDs), have a relatively low potential for pain relief caused by cancer or surgical incisions [18], there could be favourable side effects regarding cancer progression. A state of chronic inflammation can, on the one hand, contribute to the development of the tumour and, on the other hand, can be caused by the tumour and its micromilieu to create an environment that stimulates immunosuppression and evasion, as well as the proliferation of cancer cells [19]. NSAIDs, especially aspirin, have been shown to be a preventive factor in the development of various cancer entities [20]. A cyclooxygenase (COX) inhibition leads to anti-inflammatory effects, an enhancement in the immune response [21] and a platelet aggregation inhibition, which is important for the mechanism of forming metastases [22].

A phase II randomized controlled trial (RCT) showed an improvement in metastatic biomarkers of breast cancer after the implementation of propranolol and etodolac as a COX-2 inhibitor for 11 days perioperatively [23]. Another randomized study measured the apoptotic rate in squamous cell carcinoma cells of the oesophagus in patients who either received the COX-2 inhibitor meloxicam 2 weeks prior to surgery or not. They found a significant increase in apoptotic cells in the resected tumour tissue of patients receiving meloxicam (6.2% vs. 1.2%, *p* = 0.0005) [24]. In further randomized controlled trials, celecoxib was effective in preventing colorectal adenoma recurrence [25] and rofecoxib inhibited angiogenesis in colorectal cancer liver metastasis [26].

There is one prospective, randomized controlled trial evaluating the postoperative recurrence rate 45 months after rectal cancer surgery. Celecoxib was compared to a placebo and reduced postoperative inflammation, but did not affect recurrence [27] (Table 1).

The possible anticancer benefits of a perioperative NSAID use are theoretical so far. There is no evidence yet to improve survival or recurrence rates, so the use of NSAIDs should be limited to pain therapy at this time.

### Opioids

Opioid receptors are not only expressed in the central nervous system to regulate pain perception but also occur on immune [28] and tumour cells [29]. Therefore, the effects of opioids on immune and tumour cells were investigated. It has been shown that different opioids alter the natural killer cell cytotoxicity (NKCC) in different manners. For example, it was reduced by morphine and fentanyl, increased by tramadol and buprenorphine did not affect it. This was shown in vivo and in patients undergoing surgery [30]. Other immune functions affected by opioids include T cell proliferation, cytokine and antibody production and phagocytosis [31] (Fig. 1). On the tumour cells themselves, a μ-receptor activation leads to a phosphorylation of the epidermal growth factor receptor, resulting in an induced proliferation and invasion [32]. In line with this, naltrexone, as a μ-receptor inhibitor, was able to suppress DNA synthesis and reduce the number of tumour cells and angiogenesis in vivo [33]. A special interest aroused for methadone, which was found to increase apoptosis and chemosensitivity of leukaemia and glioblastoma cells in vitro and in vivo via a cyclic adenosine monophosphate (cAMP) reduction, which leads to a caspase activation [34, 35]. However, these preclinical findings have not been translated into well-designed clinical studies yet, and the adverse effects of methadone, especially in patients without pain, should be considered with caution [36].

The clinical data to assess the effects of opioids themselves on cancer progression are limited. Especially intraoperatively, multiple factors influence each other, the immune response and the tumour cells themselves. A comparison between local anaesthesia and opioids may provide clues as to which technique is beneficial, but it does not determine whether it is a reduction in negative side effects by avoiding opioids or a positive influence of local anaesthetics.

Interestingly, one study on the intraoperative dose of opioids showed significantly improved overall and recurrence-free survival in patients receiving a high dose (> 710 μg fentanyl equivalent) during the resection of squamous carcinoma of the oesophagus [37]. In other cancer types, such as oral or colorectal cancer, the intraoperatively administered opioid dose was not significantly associated with the overall or recurrence-free survival [38] or the reviewed amount of data was inconclusive [39].

The analyses of postoperatively administered opioids have fewer confounding factors, but the subject is not feasible for a prospective design. A retrospective analysis found no correlation between the total amount of consumed opioids after curative lung cancer resection and long-term survival or recurrence rates [40]. Similar results were found in two large cohort studies on postoperative chronic opiate use after breast cancer surgery [41, 42]. However, a further retrospective study of a single centre showed a significant reduction in overall survival. They compared no opioid use at all after lung cancer resection with any kind of usage [43].

Although the experimental evidence on cancer-promoting effects of opioids exists, the definite clinical verification is still lacking. Data on both intraoperative and postoperative opioid administration are inconclusive. Randomized controlled trials on whether opioids should be obtained intraoperatively or not are not practicable. Therefore, large cohort studies are the best evidence so far that the dose and choice of opioids does not worsen the cancer-related outcome of surgery.

### Local anaesthesia

Regional anaesthetic techniques are used either to avoid general anaesthesia (e.g. spinal anaesthesia, brachial plexus, distal ischiatic or femoral nerve block) or to complement perioperative pain therapy (e.g. epidural pain catheter, paravertebral block, nerve catheters or intraoperative intravenous lidocaine infusion). On the one hand, an opioid sparing effect could positively affect tumour progression; on the other hand, the anti-inflammatory and anti-adrenergic effects of local anaesthetics could play a relevant role. The most frequently discussed is the use of an epidural pain catheter in that matter.

#### Epidural anaesthesia

An immunoprotective effect of epidural anaesthesia in comparison to general anaesthesia was first described in 1980 [44]. The first retrospective studies evaluating the potential benefits for overall survival appeared more than 20 years later [45, 46] and aroused an increased interest in the subject. A meta-analysis in 2013 included fourteen studies, both retrospective and prospective, and found no association between cancer recurrence and epidural anaesthesia, but an advantage in overall survival, especially in colorectal cancer [47]. Focusing on colorectal cancer, a retrospective single-centre study showed an improved 5-year survival from 54 to 62% (*p* = 0.001) in patients with an additional epidural catheter [48]. A systematic meta-analysis with five studies concerning colorectal cancer without metastasis confirmed a positive association: HR 0.81 (95% CI 0.68–0.96, *p* = 0.055) [49]. Another study described the same benefits for prostate cancer, but not for colorectal or other cancer types [50]. Although there are many studies, including meta-analysis and systematic reviews, the results are conflicting, and a variety of limitations and bias apply.

A meta-analysis of only randomized controlled trials (RCTs) with mortality as the primary or secondary endpoint (*n* = 2201) showed an improvement in mortality rates from 4.9 to 3.1%. Further benefits for postoperative morbidities such as PONV, respiratory or cardiac complications have been shown [51]. In contrast, one of the recent randomized controlled trials showed an improvement in cellular immunity (cluster of differentiation (CD)3+, CD4+, CD4+/8+ T cells), but no difference in 3-year overall survival in gallbladder cancer [52] (Table 1).

Regarding cellular mechanisms, a prospective randomized analysis of tumour-infiltrating lymphocytes in adenocarcinoma of the lung showed an increased CD8+ lymphocyte subset and a decrease in regulatory T cells in the histology of resected tumours in patients with a combined anaesthesia (general + epidural) in comparison to general anaesthesia alone [53] (Fig. 1). Accordingly, a combined anaesthesia seems to reduce the body’s inflammatory response [interleukin (IL)-1, interleukin-8, c-reactive protein (CRP), tumour necrosis factor (TNF)α] following surgery and has a trend towards an improved recurrence-free survival in NSCLC (15 vs. 23 months, *p* = 0.08) [54] (Table 1). Similar results are shown in gastric cancer. Epidural anaesthesia preserves CD3+ cells, limits the release of IL-4 and IL-6 and increases the release of interferon (INF)-γ [55]. Two RCTs evaluate the effect of an epidural anaesthesia in combination with a TIVA for oesophagectomy vs. TIVA and intravenous opioids. Both revealed a reduced stress response with the use of an epidural anaesthesia [55, 56]. In detail, a reduction of IL-6, norepinephrine, cortisol, and adrenocorticotropic hormone (ACTH) was shown in combination with a higher secretion of IL-10, 3 h after surgery [57]. These results could not be confirmed in colorectal cancer [58].

In the past, many studies have been conducted with contradictory results. If we give more weight to the prospective randomized controlled trials, the evidence supports the use of epidural anaesthesia to increase overall survival. New studies show a preserved immune function during the critical perioperative period, which is approaching an explanation for the beneficial effects of epidural anaesthesia in some cancer types.

#### Intravenous lidocaine

Local anaesthetics, if administered epidurally, are partially absorbed into the blood. In this, they reach concentrations of 1–10 μM. As a substitute for epidural anaesthesia, the continuous application of intravenous lidocaine was therefore perioperatively investigated [59]. It has been shown that this can reduce opioid consumption and also positively influences PONV and the restoration of intestinal peristalsis after major abdominal, urological or gynaecological interventions.

This concentration of local anaesthetics is delivered into the blood flow of the tumour as well. In vitro data showed a dose-dependent antiproliferative effect of local anaesthetics on various cancer types. For example, an inhibition of migration, invasion and progression of colorectal cancer cells in vitro for lidocaine (10 μM), ropivacaine (10 μM) [60] and bupivacaine (1 mM) [61] was found. Similar results have been found in gastric cancer. Low concentrations of bupivacaine (10 μM) reduced the migration of gastric cancer cells, while high concentrations (1 mM) also increased apoptosis [62].

In vivo lidocaine (1.5 mg/kg bolus + 2 mg/kg/h) in combination with a sevoflurane narcosis significantly reduced lung metastasis of breast cancer in mice. This effect did not appear in combination with a ketamine-xylazine narcosis [63].

A retrospective analysis of intraoperative intravenous lidocaine infusion in pancreatic surgery (*n* = 915 in each group) revealed an improvement in overall survival after 1 (68% vs 62.6%, *p* < 0.001) and 3 years (34.1 vs 27.2%, *p* = 0.011) [64]. A prospective RCT identified a reduction of myeloperoxidase, histone H3 and matrix metalloproteinase MMP3 via intraoperative intravenous lidocaine infusion during breast cancer surgery. These findings support the hypothesis of an anti-metastatic effect of lidocaine [65].

The current evidence supports the use of intraoperative intravenous lidocaine infusions as a supplement in pain therapy when epidural anaesthesia is not possible or wanted. In addition, the hypothesis of an anticancer effect of lidocaine has been formulated but benefits in terms of survival and recurrence rates have yet to be demonstrated in prospective randomized controlled trials.

## Patient blood management

Cancer is a consuming disease that often results in a state of cachexia, malnutrition and anaemia. Some of the curative operations cause high blood loss, which urges the anaesthesiologist to replace the losses with red blood cell transfusions to maintain a functioning cardiovascular system. It is well known that allogeneic red blood cell transfusions are negatively associated with the tumour outcome, with the need to investigate the alternatives. Especially in orthopaedic surgery, it is well established to optimize haemoglobin levels through preoperative iron supplementation and the intraoperative use of cell saving systems. In cancer surgery, the fear occurs to enhance tumour growth via iron supplementation [66] or erythropoietin and re-transfuse circulating tumour cells via cell salvage [67]. The actual evidence for these concerns is limited and therefore needs to be reassessed as tumour patients could benefit from these alternatives to avoid allogeneic transfusions.

### Transfusions

There are meta-analyses (mostly retrospective data) concerning the impact of allogeneic blood transfusions on cancer outcome for almost all main tumour entities. To name a few, the odds ratio for all-cause mortality in gastric cancer is 2.17 [95% CI 1.72, 2.74, *p* < 0.001] when allogeneic blood transfusions were received [68]. For hepatocellular carcinoma, the risk ratio for mortality decreases continually from 0.9 [95% CI 0.87, 0.93, *p* < 0.05] after 1 year to 0.62 [95% CI 0.48, 0.8, *p* < 0.05] after 10 years if no blood transfusions were given [69]. Similar results in prostate cancer, where a hazard ratio of 1.43 [95% CI 1.24, 1.64, *p* < 0.01] for overall survival and even 1.74 [95% CI 1.18, 2.56, *p* = 0.005] for cancer specific survival was evaluated [70].

It has also been shown that the amount of transfusions is relevant for the prognosis of cancer outcome. A meta-analysis of ampullary cancer, operated as a pancreaticoduodenectomy, identified a threshold of ≥ 3 units of blood to be associated with a worse outcome than < 3 units or no transfusion [71]. A similar threshold of 800 ml was found in the above-mentioned study on gastric cancer [68].

The safety of blood salvage in cancer patients has been under review for over a decade now. There are experimental and small clinical studies that indicate that no increased recurrence or mortality rates occur from cell salvage when a leuco-reduction filter is used [72]. A meta-analysis comparing intraoperative blood salvage and allogeneic blood transfusion in malignant diseases in general revealed no difference regarding overall survival or recurrence rate [73] (Table 1). The German cross-sectional guidelines for hemotherapy recommend an autotransfusion of saved wound blood for tumour patients after a radiation with 50 Gray [74].

In conclusion, allogeneic blood transfusions should be avoided or limited as much as possible. There is currently no evidence that intraoperative blood salvage should be preferred over transfusions in cancer surgery, but it has been proven to be a safe alternative if allogeneic transfusions are either rejected by the patient or limited in their availability due to rare blood types or transfusion-related antibodies.

### Iron supplementation

The exact prevalence of a total or functional iron deficiency causing anaemia varies between the different tumour types but concerns on average 36% and is a constant distress towards the physical abilities and quality of life of cancer patients [75]. In mild cases of anaemia, oral supplementation can be considered whereas in moderate or severe cases, the intestinal uptake of iron is so impaired that intravenous supplementation is necessary [76]. Although the haemoglobin levels and consequently the quality of life and fitness of different cancer patients could be improved especially by intravenous iron supplementation, the usage is small [77]. A systemic review in 2015 evaluated seven studies concerning the efficiency of preoperative iron supplementation to treat cancer specific anaemia. They found a reduction of allogeneic blood transfusions in all studies [78]. After that, studies with both conclusions were conducted but only concerning colorectal surgery. Mostly retrospective studies found a reduction of transfusions [79, 80], whereas a randomized controlled trial could not find any difference in the transfusion rate after oral or intravenous iron supplementation [81]. A prospective multicentre observational trial included all kinds of surgical patients and screened them for anaemia and iron deficiency. Iron supplementation was given intravenously in iron deficiency, with or without anaemia, 1, 2 or more than 2 weeks prior to surgery. Red blood cell transfusions could be reduced postoperatively in all patients, but only intraoperatively in those patients who received the infusion more than a week before surgery [82].

Experimental studies proved the important role of iron in the cell cycle, angiogenesis and formation of metastasis founding the hypothesis of a pro-oncotic effect of iron [67]. Contrarily, a high iron load in the tumour environment of non-small cell lung cancer tissue was associated with an increased M1 polarization of tumour-associated macrophages (TAM). TAMs are either polarized towards a pro-inflammatory type M1, or a type M2 associated with suppressing immune activity and promoting proliferation and angiogenesis [83]. Therefore, an increase in M1 macrophages leads to an increased pro-inflammatory environment and thus a survival benefit in patients with high iron levels in adenocarcinomas: HR 0.298 (95% CI 0.112; 0.790 *p* = 0.015) [84].

Epidemiological studies show conflicting results regarding a correlation between iron levels and carcinogenesis. An observational study on multiple myeloma identifies an iron overload as well as an iron deficiency as a factor for a worsened overall and recurrence-free survival [85]. There is one retrospective cohort study showing no decrease of the 5-year overall survival or progression-free survival by a preoperative intravenous iron supplementation of 1000–2000 mg in colorectal cancer patients undergoing radical resection [86].

In summary, the experimental and clinical data about the relevance of a preoperative iron substitution to treat anaemia is conflicting. Although a cancer-promoting effect of iron exists in some experimental studies, there are also data supporting a beneficial effect of a positive iron status at least for pulmonary adenocarcinoma. There is still no clinical evidence that a preoperative iron supplementation negatively affects survival or progression of cancer. However, since there is mixed evidence about the ability of iron supplementation to reduce allogeneic blood transfusions, it cannot be recommended to expend the preoperative substitution of iron in general. But this alternative could again be relevant for people who refuse blood transfusions or cannot receive them for other reasons.

## Intraoperative oxygen application

There are numerous studies evaluating the intraoperative fraction of inspired oxygen (FiO_2_) regarding surgical site infections, wound healing and anastomotic insufficiencies or respiratory complications. But the data on an association towards tumour progression is limited. On the one hand, tumours are faced with hypoxia due to a poor vascularization. The tumour cells and their microenvironment are adapted to this niche with an enhanced production of growth mediators. On the other hand, high oxygen levels are associated with angiogenesis, erythropoietin production and oxidative stress, all factors also in favour of the tumour.

Experimental studies implied a tumour-suppressive effect of hyperbaric oxygen [87] and, also, normobaric hyperoxia via increased apoptosis rate of tumour cells in vitro and in vivo [88].

One clinical study, the PROXI trial, compared a FiO_2_ of 80% vs. 30% intraoperatively and 2 h postoperatively for abdominal surgery and originally evaluated that there is no difference in surgical site infections, but a higher 30-day mortality with 80% FiO_2_. There is a follow-up regarding the mortality rate after a medium of 2.3 years, showing a higher mortality for 80% FiO_2_ (23.2% vs. 18.3%), only significant in cancer patients (HR 1.45, *p* = 0.009) [89].

In summary, there are contradictory results from experimental and clinical studies that suggest that an elevated intraoperative oxygen fraction should be used with caution until further research has been carried out. Until then, a FiO_2_ around 30% should be preferred.

## PONV prophylaxis

Postoperative nausea and vomiting (PONV) occur with an incidence of 20–40% after general anaesthesia and cause a high level of distress in many patients [90]. There are standard protocols to prevent or limit the severity of PONV, including an intraoperative application of 0.1 mg/(kg bodyweight) dexamethasone and 10 μg/(kg bodyweight) granisetron. It is known that glucocorticoids such as dexamethasone inhibit the immune system, raise blood glucose levels and have anti-inflammatory effects, thus promoting further tumour growth.

There are three retrospective studies that evaluate whether there is any correlation between a single dose of dexamethasone intraoperatively and a worsened tumour outcome. These studies included breast, colorectal and pancreatic cancer, and none could find a negative impact of a single dose of dexamethasone on mortality or recurrence. On the contrary, positive effects like a reduced rate of infectious complications and systemic inflammatory reactions were described in colorectal and pancreatic surgery [91–93].

In respect of these results, the standard PONV prophylaxis should be used on cancer patients at increased risk of developing nausea and vomiting.

## Conclusion

The operation of a malignant tumour is a critical part to determine the long-term outcome of cancer patients. Besides the resection of the primary tumour, any alterations in the immunological abilities of the body to destroy remaining local and circulating tumour cells as well as direct influences of anaesthetics on these cells should be considered. Perioperative choices in anaesthesia include inhalational vs. intravenous narcotics, the application of different opioids and peripheral analgesics like NSAIDs, the addition of regional anaesthesia during surgery, patient blood management, the intraoperative oxygen application and the supplementation of dexamethasone to prevent nausea and vomiting.

The choice of narcotics might play a role in certain types of cancer. Propofol has direct antiproliferative effects on the tumour cells and preserves the immunological function and enhances chemosensitivity in vitro and in vivo as indirect effects on cancer progression. In patients undergoing surgery, a reduction of VEGF and TGF-β was found as possible mechanisms to diminish tumour growth. Propofol with its anti-inflammatory abilities might be superior to inhalational agents regarding the survival of, e.g. colorectal or lung cancer patients. It does not show these beneficial effects in breast cancer. More prospective randomized trials are ongoing. Hopefully, their results can lead to more definite recommendations in this topic.

Concerning cancer pain management, NSAIDs have anti-inflammatory and anti-thrombogenic properties which proved to be an advantage in the primary prevention of tumours, especially colorectal. Their role in the perioperative period and therefore in the secondary prevention of recurrence is understudied and needs further investigation. Opioids as the next step in pain therapy interact with the patient’s immune system. Opioids like fentanyl and morphine inhibit the natural killer cell cytotoxicity, whereas tramadol improves it. A direct induction of proliferative signalling pathways via the μ-receptor might influence tumour growth and survival. But clinical studies cannot prove a negative impact of intraoperative or postoperative opioid doses on the survival. Therefore, opioids remain the substantial foundation of cancer pain therapy.

Epidurally and intravenously administered local anaesthetics have favourable effects on overall survival of cancer patients. They have been shown to induce tumour-infiltrating cytotoxic (CD8+) lymphocytes and inhibit tumour-promoting regulatory T cells. Also, the immune components in the blood are changed towards an enhanced activation of the adapted immune system (CD3+, CD4+, CD8+) and a reduced systemic inflammatory reaction (reduction of Il-1, Il-4, Il-6, Il-8, TNFα, CRP; increased release of Il-10, INFγ) and stress response (reduced cortisol and norepinephrine levels). Procedures with a high amount of postoperative pain like open pancreatic or liver surgery should be covered with additional epidural pain catheters, whereas patients with shorter and less painful procedures can profit from intravenous lidocaine infusion as an alternative.

Allogeneic blood transfusions should be avoided or used as little as possible as transfusions were shown to significantly worsen the overall survival of cancer patients throughout the different types of cancer. Additionally, to no transfusions at all, a threshold of 3 units or 800 ml of transfusions was identified as a prognostic marker for the outcome. Preoperative iron supplementation or irradiated intraoperative cell salvage is an alternative in specific cases.

Intraoperative oxygen application should be limited to assure a normal partial pressure of oxygen in arterial blood (P_a_O_2_) and oxygen saturation (SpO_2_). Contradictory to experimental results, a higher oxygen fraction (e.g. 80%) was associated with an increased 30-day and long-term mortality in cancer surgery.

Dexamethasone as an intraoperative single shot is considered a safe method to prevent postoperative nausea and vomiting.

Considering the numerous effects and interactions from anaesthetic agents with the tumour environment and the immunological function, anaesthesia is an important influence in tumour progression. Further research is necessary to formulate recommendations specific for the different tumour entities.

## Data Availability

The data that support the findings of this study are openly available in MEDLINE® at https://pubmed.ncbi.nlm.nih.gov/
